# Exploring Challenges in Conducting E-Mental Health Research Among Asian American Women

**DOI:** 10.31372/20180304.1016

**Published:** 2018

**Authors:** Andrew Thomas Reyes, Rose E. Constantino, Rogelio A. Arenas, Judzia N. Bombard, Alvin Ryan Acupan

**Affiliations:** a University of Nevada, Las Vegas, Las Vegas, NV, USA; b University of Pittsburgh, Pittsburgh, PA, USA

**Keywords:** e-mental health, e-mental health research, Asian American women

## Abstract

In this discussion paper, we explore the challenges of conducting e-mental health intervention research among Asian American women and propose a model for addressing these barriers. Based on an extensive literature review, we identify two main types of barriers to conducting e-mental health intervention research among Asian American women: recruitment barriers and adherence barriers. Recruitment barriers are further subcategorized into those related to (1) stigmatized cultural beliefs about mental illness and mental health services; (2) lack of awareness about mental health services; and (3) language barrier. As to adherence barriers, the two identified subtypes concern (1) acuity and severity of mental health condition; and (2) lack of time. In order to enhance recruitment and adherence in e-mental health intervention research among the studied population, we formulate the following three main research strategies, namely: (1) considering the cultural and social contexts of Asian American women in the development of e-mental health interventions; (2) determining appropriate program length; and (3) conducting feasibility studies to test e-mental health interventions. We suggest that nurse researchers integrate our proposed model in conducting e-mental health interventions among Asian American women. Our proposed model also implies that nurses play an important role in encouraging Asian American women’s acceptance of and adherence to e-mental health interventions. In order to overcome the obstacles to conducting e-mental health research among Asian American women, we recommend that nurses familiarize themselves with credible, relevant, and evidence-based e-mental health resources and integrate online mental health services and information within their nursing practice.

In recent decades, the ever-growing demand for mental health services has started to exceed the number of available mental health professionals, resulting in limited options for individuals seeking access to medication prescriptions and referral to specialists (Mar et al., 2014). In this context, a rapid growth of e-mental health research offers a promise of increased access to mental health care, particularly for those who would not otherwise access face-to-face treatments and programs (Lal & Adair, 2014). Accordingly, e-mental health services can serve as an alternative to the conventional face-to-face mental health care services.

By definition, e-mental health services embrace programs, resources, and tools using the Internet and related information technologies (i.e., informative websites, web-based therapies, smartphone applications, social media, and videotelephony) used for mental health promotion and symptom prevention, as well as for management of mental disorders and psychological problems, and delivered with or without human support (Barak, Klein, Proudfoot, 2009; Riper et al., 2010). Relevant examples of e-mental health services and resources include mental health smartphone applications, web-based psychological interventions with or without therapist support, and online counseling. However, despite the rapid growth of these tools and resources in the healthcare market, most of them have not been empirically tested (Donker et al., 2013). For instance, while Ben-Zeev et al. (2013) reported that, as of 2013, there were over 10,000 smartphone apps related to health that were available for download, only five of these apps, according to Donker et al.’s (2013) systematic review, were empirically tested in the same year. Furthermore, many of these e-mental health tools are constantly changing through revisions and updates, which poses certain challenges when these tools are tested in clinical trials. Finally, several reports have noted a lack of methodological rigor of these e-mental health tools (e.g., Clough & Casey, 2015). In sum, the factors outlined above make it imperative to thoroughly explore feasibility, acceptability, efficacy of, and adherence to e-mental health resources through intervention research.

Furthermore, it is equally important to explore the relevance and applicability of e-mental health services to ethnically and racially diverse target population groups, such as the U.S. population. However, a major limitation of previous research on e-mental health tools is that relevant studies did not target specific ethnic and racially diverse groups; moreover, many of those studies did not report ethnicities of the participants. For example, most studies included in Meurk, Leung, Hall, Head, and Whiteford’s (2016) systematic review of e-mental health services for depressive and anxiety disorders did not report demographic data on ethnicity. Yet, as argued by Bennett-Levy, Singer, DuBois, and Hyde (2017), in order to increase the efficacy and user uptake of the e-mental health tools, those tools need to be culturally relevant for the studied populations. On a large scale, ethnic-specific prevention and treatment programs need to be promoted as possible solutions to reduce inequities in mental health service utilization among ethnic minority populations. In support of this argument, Takeuchi, Sue, and Yeh (1995) found that ethnic-minority individuals attending ethnic-specific mental health programs had significantly higher rates of mental health service use than those ethnic-minority clients who accessed mainstream services.

Asian Americans currently represent the fastest growing racial group in the United States (U.S. Census Bureau, 2017). Yet, compared to other major racial groups in the U.S., this population group was reported to have lower rates of seeking mental health treatment (Lee, Martins, Keyes, & Lee, 2011; Wong, Collins, Cerully, Seelam, & Roth, 2017). For instance, a national study of Asian Americans diagnosed with a mental disorder found that only 28% of the respondents used specialty mental health services (Le Meyer, Zane, Cho, & Takeuchi, 2009). Asian American women, as a major subgroup of Asian Americans, were reported to have the highest increased rate of suicide from 2009 to 2014 compared to their Black and Hispanic counterparts (Curtin, Warner, & Hedegaard, 2016). According to recent estimates, suicide is the second leading cause of death among Asian American women between the ages of 20 to 24 (Center for Disease Control and Prevention, 2013). Likewise, in a study of Asian American women, Augsberger, Yeung, Dougher, and Hahm (2015) found that 43% of the participants had current moderate to severe depressive symptoms or a lifetime history of suicidal ideation or suicide attempt. Additionally, more Asian women suffer from depression and more severe symptoms of depression than their male counterparts (David, 2008; Suen & Morris, 2006; Ying & Han, 2006). What aggravates the situation is that Asian American women are not only at high risk for developing severe symptoms of mental disorders and psychological distress, but also underuse professional mental health care (Augsberger et al., 2015). For instance, evidence is available showing that Asian American women have lower rates of seeking mental health services than their White American counterparts (Appel, Huang, Ai, & Lin, 2011).

The above-mentioned prevalence of mental disorders among Asian American women, their high risk for developing severe mental disorder symptoms and psychological distress, as well as their underutilization of mental health services make it imperative to explore alternative mental health resources that this population group may likely access and use. Among such alternative mental health resources are e-mental health services.

To date, however, empirical research on acceptability and utilization of e-mental health services among Asian American women remains scarce. Therefore, in order to bridge this gap in the literature, it is important to establish a foundational basis for planning research processes that would help increase recruitment and adherence of e-mental health interventions among Asian American women. Accordingly, this discussion paper aims to provide a preliminary groundwork based on the available empirical literature in addressing the challenges, barriers, and strategies of conducting e-mental health intervention research among Asian American women. To this end, we focused on two aspects of e-mental health intervention research: recruitment and adherence. In order to accomplish this preliminary groundwork, we first reviewed previous research on the utilization of mental health services (both face-to-face and e-mental health interventions) among Asian Americans. Thereafter, we explored the barriers to conducting e-mental health intervention research among existing e-mental health studies not specifically targeted to Asian American women (since there is limited empirical literature on e-mental health intervention research among Asian American women). On synthesizing these two areas of the literature review, we identified barriers to and strategies in conducting e-mental health intervention research among Asian American women. Figure 1 shows our proposed framework for exploring challenges in conducting e-mental health research among Asian American women.

**Figure 1:**
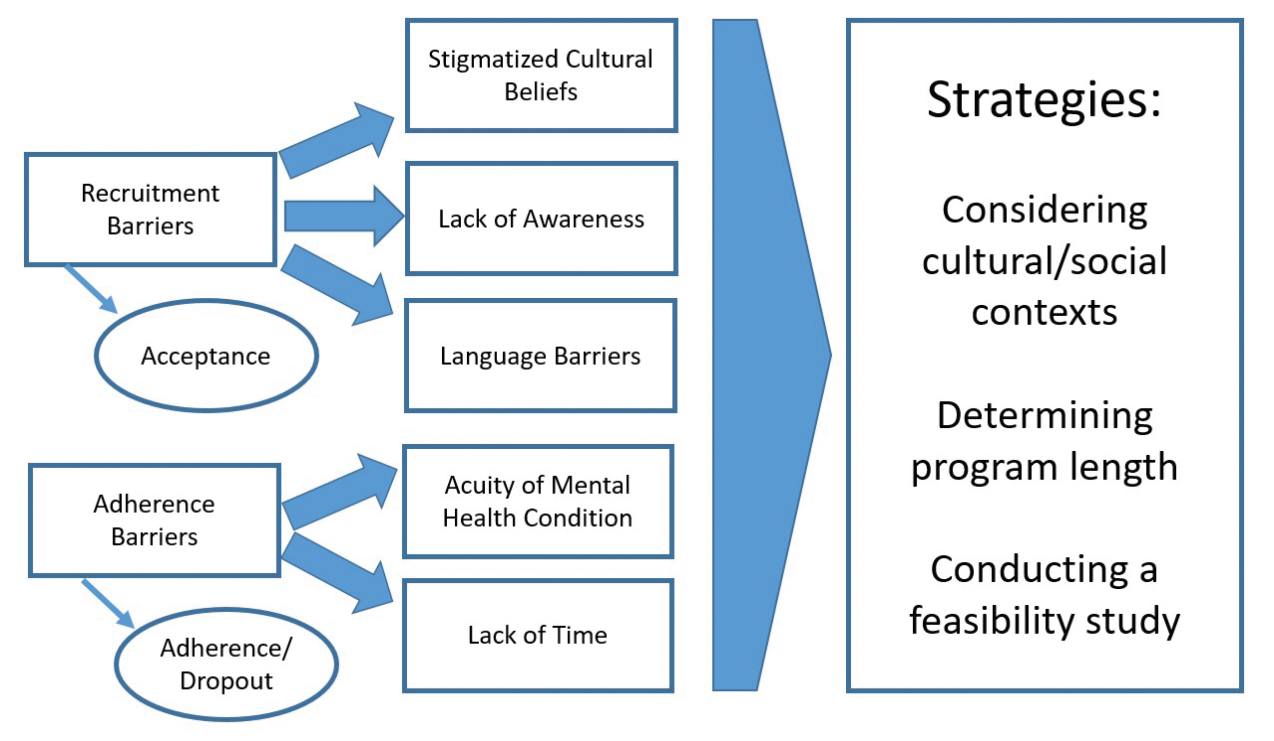
Proposed framework for exploring the challenges of conducting e-mental health research among Asian American women.

Overall, there are two main types of barriers to conducting e-mental health intervention research: recruitment and adherence barriers (see Figure 1). In the category of recruitment barriers, we identified (1) stigmatized cultural beliefs about mental illness and mental health services; (2) lack of awareness about mental health services; and (3) language barrier (see Figure 1). In the category of adherence barriers, we propose to differentiate between the barriers related to (1) acuity and severity of mental health condition and (2) lack of time (see Figure 1). A key concept and/or variable that should be explored in determining recruitment barriers is *acceptance*, while the key concepts and/or variables for investigating adherence are *adherence* and *dropout* (see Figure 1). In order to address the identified barriers to conducting e-mental health intervention research among Asian American women, we formulated the following three key recommendations for the design, development, and testing of e-mental health interventions: (1) considering the cultural and social contexts of Asian American women in the development of e-mental health interventions; (2) determining appropriate program length; and (3) conducting feasibility studies (see Figure 1).

The remainder of this discussion paper is structured as follows. On an in-depth discussion of the two types of barriers and their subtypes, we formulate and discuss recommendations for addressing the barriers to conducting e-mental health intervention research among Asian American women. This is followed by a discussion of theoretical and practical implications of the proposed model on exploring the challenges of conducting e-mental health research among Asian American women.

## Barriers to Conducting E-Mental Health Intervention Research

### Recruitment Barriers

The first category of barriers we identified in relation to conducting e-mental health intervention research among Asian American women are recruitment barriers. These barriers relate to the factors affecting the successful rates of recruiting Asian American women as participants in e-mental health intervention research. In essence, these barriers are based on the phenomenon of *acceptance,* which is operationally defined as the intention to use a technology (Venkatesh, Morris, Davis, & Davis, 2003). In the context of e-mental health intervention research among Asian American women, the phenomenon of acceptance refers to these women’s intention to use an e-mental health intervention.

Overall, the acceptance of mental health services and e-mental health tools contributes to the increased participant recruitment and uptake of e-mental health interventions. For example, several studies (albeit not specific to Asian Americans) demonstrated that, while the severity of symptoms propels one to seek professional mental health assistance, individuals tend to participate in e-mental health research because of their acceptability and openness to e-mental health services (Fonseca, Gorayeb, & Canavarrio, 2016; Klein & Cook, 2010; Waller & Gilbody, 2009).

At present, however, research on the acceptance and intention to use e-mental health interventions by Asian Americans is rather limited; moreover, most of these studies involve both male and female Asian American participants. Yet, what underscores the importance of e-mental health interventions for specifically Asian American women is that, according to recent evidence, significantly more Asian Americans own and use digital technology than other racial/ethnic groups (Rainie, 2011). For instance, a study on Asian Americans’ ownership and usage of digital technology demonstrated that about 60% of Asian Americans (more particularly, Filipinos and Koreans) downloaded at least one mobile app as compared to Latinos of only about 43% (Bender et al., 2014). However, Bender et al. (2014) did not report specific data about Asian American women; in addition, most of the mobile health apps discussed in Bender et al.’s (2014) study were related to fitness, and mental health apps were not reported.

In reviewing the literature, we identified three subcategories of recruitment barriers that prevent Asian American women from participating in e-mental health intervention research: (1) stigmatized cultural beliefs about mental illness and mental health services, (2) lack of awareness about mental health services, and (3) language barrier. In what follows, these three types of recruitment barriers are discussed in further detail.

#### Stigmatized cultural beliefs

Stigmatized beliefs about mental illness and mental health services may present a barrier for recruitment to and acceptance of e-mental health intervention research among Asian American women. Overall, our review of the literature suggests that there are three aspects of stigmatized beliefs related to the Asian culture that contribute to hindering recruitment and acceptance of e-mental health intervention research.

First, among the culture-specific aspects of Asian American women’s stigmatized cultural beliefs against mental illness and seeking mental health help is the cultural value of “saving face.” This cultural value, which pertains, among other issues, to downplaying and concealing mental health problems in order to prevent shame in their families, is particularly salient among Asian cultures (Gong, Gage, & Tacata, 2003). The collectivistic nature of many Asian cultures creates a propensity to de-prioritize individual needs (e.g., a person’s need to seek specialized mental health services) to the needs of the family in order to protect the family’s reputation (Sawrikar & Katz, 2017). Therefore, while the communal nature of relationships among Asian families provides social support, the concealment of mental health problems within families in order to preserve their social integrity can present a barrier to accepting e-mental health intervention research, thereby impeding the e-mental health recruitment process. Asian American women, as compared to their male counterparts, may become particularly unwilling to accept e-mental health intervention research because, for them, the family is the basic unit of how they define themselves (Meyer, Dhindsa, & Zane, 2012). Therefore, disclosing their mental health problems outside of their families and being publicly associated with mental health services and related research programs present a threat to their family integrity and reputation. In support of this contention, several previous studies demonstrated that Asian Americans’ perceptions of loss of “face” are significantly negatively correlated with the intention to seek professional mental health assistance (e.g., Gong et al., 2003; Leong, Kim, & Gupta, 2011). Based on this evidence, we speculate that Asian American women’s cultural value of “saving face” may also render them from fully participating in e-mental health research. Additionally, previous studies also showed that Asian American women’s cultural value of “saving face” is significantly related to acculturative stress (Leong et al., 2011; Reyes, Serafica, Cross, Constantino, & Arenas, 2018; Sung, 2010). This means that the effect of “saving face” may be different between recent immigrant and acculturated Asian American women. Therefore, the degree of Asian American women’s acculturation is another variable to be considered in exploring how the cultural value of “saving face” might influence Asian American women’s acceptance of e-mental health interventions.

The second stigmatized cultural belief—or rather, a constellation of cultural beliefs—specific to Asian American women that may present as a barrier to recruitment is their cultural conceptions of mental illness and related treatment. In Eastern mind-body holistic framework where the distinction between physical and psychological illnesses is blurred (Chan, Ho, & Chow, 2002), many Eastern and Asian cultures advocate indigenous causes of mental illness. This lack of differentiation between physical distress and mental/emotional distress in many Asian cultures results in somatic representation and expression of psychological symptoms. Accordingly, psychological and psychiatric assistance is sought from traditional healers or physicians, rather than from mental health professionals (Chan et al., 2002; Sanchez & Gaw, 2007). Other cultural conceptions of mental illness in many Asian cultures that can contribute to Asian Americans’ sources of stigma towards mental health services (Abdullah & Brown, 2011) include supernatural and religious causes of mental illness, developing mental illness as a consequence of wrongful actions committed against one’s ancestors and God, and mental illness as a sign of weakness (Fogel & Ford, 2005; Lam, Tsang, Chan, & Corrigan, 2006; Lauber & Rössler, 2007). Serving as barriers to help-seeking behaviors towards professional, psychiatric/psychological help (Leong & Lau, 2001), these cultural conceptions impose challenges to recruitment of participants to e-mental health intervention research.

Finally, the third aspect that underlies the emergence of stigmatized beliefs about mental illness and mental health services among Asian American women is the fact that most available e-mental health tools are based on Western models of psychotherapy; hence, these e-mental health tools may be perceived as conflicting or contradictory to many Asian Americans’ notions of mental illness and related treatment. It is interesting to note, however, that Asian Americans’ acculturation promotes help-seeking behaviors towards professional psychiatric/psychological help (Leong et al., 2011). Specifically, those Asian Americans who are able to embrace both their indigenous cultural beliefs and the general belief systems of the dominant culture (i.e., the U.S. Westernized culture) tend to have more positive attitudes towards seeking help (Berry, 1997). Therefore, the degree of acculturation of Asian American women in the U.S. may have a positive impact on recruiting these women to participate in e-mental health intervention research.

#### Lack of awareness about mental health services

Another barrier to recruiting Asian American women as participants of e-mental health intervention research is their lack of awareness of mental health services. Previous studies reported that Asian Americans’ lack of awareness and knowledge about available mental health services is associated with this population’s low utilization of mental health services (Abe-Kim et al., 2007; Spencer, Chen, Gee, Fabian, & Takeuchi, 2010). This lack of awareness about mental health services and the subsequent low use of such services may be related, as suggested by Leong and Lau (2001), to economic and geographical barriers such as “having to work two jobs, unable to get time off to seek services, lack of child care, unmanageable distance to facility, [and] lack of transportation” (p. 205). Available evidence also shows that lack of utilization of mental health services among ethnic minorities is related to low recruitment for mental health research (Waheed, Hughes-Morley, Woodham, Allen, & Bower, 2015). Hence, accessing eligible research participants may be challenging if sources of recruitment are limited only to locations and resources already providing mental health services. Therefore, alternative recruitment strategies, such as using screening processes and recruiting in neutral, non-stigmatizing locations (i.e., community-based locations, social media), may need to be considered.

#### Language barrier

The third barrier to recruiting Asian American women as participants to e-mental health intervention research is the lack of proficiency in the English language. The detrimental impact of this barrier on the recruitment of Asian American women as participants to e-mental health intervention research is particularly significant when we consider that most of the existing e-mental health tools are in English (Bender et al., 2014). Indeed, previous studies convincingly demonstrated that lack of English proficiency among many Asian Americans is one of the most significant barriers to mental health service utilization (Abe-Kim et al., 2007; Kung, 2004). Furthermore, in a study on practical barriers of mental health service use among Asian Americans, Kung (2004) found a weak correlation between education and service use; instead, a significant predictive effect found in this study was specifically for the language barrier. The above underscores the importance of the language used in e-mental health interventions, and this is particularly relevant to e-mental health tools that largely rely on written instructions and verbal communication. Since most U.S. mobile mental health apps are in the English language, non-English speaking Asian Americans may refrain from accessing (i.e., downloading health apps) and using such tools.

### Adherence Barriers

The second category of barriers we identified in relation to conducting e-mental health intervention research among Asian American women are adherence barriers. These barriers relate to the factors affecting the retention of participants in the study and the completion of the intervention under study. Barriers of this type are foundationally based on two main concepts: *adherence* and *dropout*. Christensen, Griffiths, and Farrer (2009) defined adherence as the “extent to which individuals experience the content of the Internet intervention” (para 5); while dropout was operationally referred to the variable measured by how a participant “fails to complete the research trial protocol associated with an Internet intervention, and thus does not complete trial assessments” (para 5). In this paper, we define the concept of adherence as the extent to which these women use and apply the required activities and exercises within a proposed e-mental health intervention; the concept of dropout, in turn, is deemed to refer to the rate and reasons for failing to complete the intervention itself and the assessments and surveys included in the trial. Overall, based on our review of the literature, two adherence barriers can be discerned. In what follows, these barriers are discussed in further detail.

#### Acuity and severity of mental health condition

The first adherence barrier to conducting e-mental health intervention research among Asian American women is the acuity and severity of the women’s mental health condition.^1^ Overall, available evidence shows that, despite Asian American women’s experience of increased psychological distress and acuity of mental disorders, they still have a low level of willingness to use professional mental health services (e.g., Augsberger et al., 2015). For instance, a study on Chinese American mothers with postpartum depression demonstrated that, despite increased depressive symptoms, these mothers had increased resistance towards seeking professional mental health services (Wong, 2011). Furthermore, in a comparative study of Asian American and White American college students, Kim, Park, La, Chang, and Zane (2016) found that, despite a greater severity of psychological distress among Asian American college students, these students participated in significantly fewer counseling sessions than their White American counterparts. Finally, in a systematic review of previous research on domestic violence, Kim (2000) demonstrated that, despite the growing number of the cases of family battery in Asian American households, Asian American women tend to avoid seeking for mental health services.

The above-mentioned evidence is largely congruent with the findings reported in other studies that highlight the general trend—irrespective of ethnicity—that severe levels of mental disorder and psychological symptoms may decrease adherence. For instance, in a study that evaluated an online bipolar education program, Nicholas et al. (2010) found that difficulties associated with the acute phase of the disorder and avoidant behaviors (e.g., refusing to think about the illness) were among the major reasons for the lack of adherence to the intervention. Likewise, in their study on a web-based psychotherapy for posttraumatic stress disorder (PTSD), Knaevelsrud, Brand, Lange, Ruwaard, and Wagner (2015) explained that the high attrition rate was related to participants’ active PTSD symptoms, as well as to the ongoing violence in their environments, which shifted their focus from adhering to the intervention to ensuring that their day-to-day needs were attended to first. Finally, in a study of an e-mail-based self-help program for depression, Morgan, Jorm, and Mackinnon (2013) observed that the participants who were screened positive for depression at pre-intervention were less likely to have post-intervention data.

However, while severe levels of mental disorder and psychological symptoms may decrease adherence, some studies (albeit not specific to Asian Americans) demonstrated that a certain lower degree of severity of symptoms may be needed for participants to access and complete e-mental health interventions. For instance, in a systematic review of Internet interventions for anxiety and depression, Christensen et al. (2009) found that a lower severity of depressive and anxiety disorder symptoms predicted increased adherence to e-mental health interventions. Likewise, Fonseca et al. (2016) found that women who tested positive for depression screening, which indicated a need for help, reported a greater use of and more positive attitudes towards using e-mental health tools. In addition, individuals with stronger depressive symptoms, but lower personal stigma towards mental health services were reported to be more likely to adhere to Internet-based mental health intervention research (Crisp & Griffiths, 2014; but see Nicholas et al., 2010^2^).

Taken together, the studies reviewed above suggest that disease-related factors may have an impact on participants’ adherence and completion of e-mental health interventions. Specifically, although the presence of a mental disorder and/or psychological symptoms may indicate the need for help, the unpredictability of the course of the mental disorders or acute/chronic stressors that participants are experiencing outside the research study context may negatively influence their adherence to e-mental health research. Therefore, in the design and development of e-mental health interventions, Asian American women’s acute phase and severity of depressive and anxiety symptoms should be taken into consideration.

#### Lack of time

The second adherence barrier to conducting e-mental health intervention research among Asian American women is the participants’ lack of time to complete the e-mental health intervention research study. Although this specific barrier is not exclusive to the Asian American population, previous e-mental health studies involving the general population highlighted time constraints to be among the major factors related to low adherence and increased dropout rates (e.g., Carlbring et al., 2006; Warmerdam, van Straten, Twisk, Riper, & Cuijpers, 2008). The reasons underlying Asian American women’s lack of time to complete required tasks and assessments in e-mental health intervention research include time constraints imposed by these women’s everyday life, such as employment and family commitments (e.g., Leong & Lau, 2001).

## Recommendations for Addressing the Barriers

In order to address the recruitment and adherence barriers to conducting e-mental health intervention research among Asian American women, we propose the following three key recommendations: (1) considering the cultural and social contexts when developing interventions; (2) determining appropriate program length; and (3) conducting feasibility studies in order to investigate optimal recruitment strategies, adherence, and dropouts. In the remainder of this section, the three formulated recommendations are discussed in further detail.

### Considering Cultural/Social Contexts in Intervention Development

Given that cultural aspects of Asian cultures can, as discussed in the previous section, give rise to stigmatized views of mental health services, it is imperative to consider the cultural and social contexts of Asian American women when developing and testing e-mental health interventions. As discussed before, these stigmatized beliefs may be underpinned by collectivistic values of many Asian cultures—and particularly, by the culture-specific concept of “losing face.” Accordingly, in the development of e-mental health interventions and the design of research studies to test such interventions, we recommend considering the principles outlined in Hofstede’s (1980, 2011) framework of individualism/collectivism as dimensions of culture. The use of this framework would not entail promoting individualistic behaviors among Asian American women, but rather an integration of collectivistic values and behaviors as essential components needed to enhance participants’ recruitment and adherence to e-mental health research. For example, if during the conduct of an e-mental health research study, Asian American women are demonstrating early signs of lack of adherence (e.g., not completing tasks in an intervention related to lack of time), study investigators should recognize that it is likely that the participants’ collectivisitic values (i.e., their tendency to prioritize the needs of the group over individual needs) contribute to their lack of time of completing the intervention. In the event of stressful situations within their families, Asian American women would tend to prioritize the welfare and integrity of their families over their personal needs as individuals, which may result in a low adherence and an increased number of dropouts. Therefore, strategies in increasing adherence should integrate principles of collectivism rather than addressing lack of time related to completing the study as a personal or individual issue of the participant.

Moreover, in the development of culturally relevant e-mental health interventions, there should be a balance in the provision of both support and connection (from professional providers/therapists and peers) and privacy/anonymity. In essence, anonymity and privacy are integrated into e-mental health research so that to prevent, or at least minimize, personal stigma associated with the use of e-mental health services (Crisp & Griffiths, 2014). Hence, individuals who have stigmatized beliefs about mental health services may prefer to access e-mental health services due to the privacy and anonymity these services provide (Klein & Cook, 2010; Young, 2005). However, some studies (albeit not specific to Asian Americans) show that anonymity was rated the least important factor for using e-mental health tools (Carper, McHugh, & Barlow, 2013; Musiat, Goldstone, & Tarrier, 2014).

Despite the seeming contradiction, human support and anonymity in e-mental health interventions should not to be viewed as mutually exclusive elements of e-health mental services. As demonstrated by Mar et al. (2014), e-mental health research participants valued both having the human support (provided by professionals and peers) and privacy and anonymity afforded by e-mental health interventions. This balance of human support and privacy/anonymity provision in designing e-mental health interventions is particularly relevant for Asian American women who, as discussed earlier in this paper, have stigmatized beliefs of mental illness and who might consider e-mental health interventions to be less effective as compared to face-to-face methods. Therefore, rather than dichotomizing the processes of human support and privacy/anonymity, researchers should contextualize these two processes in a continuum. Accordingly, a key consideration in designing e-mental health interventions should be how much and what type of human support is needed, and how much self-direction should be integrated into the intervention.

In general, the benefits of integrating human support into the intervention were consistently reported in the literature. For instance, several meta-analyses of e-mental health interventions (albeit not specific to Asian Americans) demonstrated that therapist-supported programs had higher levels of completion as compared to self-directed programs (Newman, Szkodny, Llera, & Przeworski, 2011; Richards & Richardson, 2012; but see Grist & Cavanagh, 2013^3^).

With regard to peer support, it was also shown to have a strong positive impact on user adherence rates (e.g., Griffiths, Calear, Banfield, & Tam, 2009). For instance, in a study of an online bipolar education program, Nicholas et al. (2010) demonstrated that the participants (albeith not specific to Asian Americans) who received email support from expert patients had higher adherence rates than those who did the program in the self-directed modality. However, in populations of Asian American women, the forms of peer support to be used in e-mental health interventions should be carefully considered and tailored to the needs of the target population. Many Asian American communities tend to be cohesive and small. Therefore, disclosing mental health issues outside their families to individuals within their ethnic communities may be perceived as a threat to their family integrity and reputation. Hence, peer support, especially from peers of the same ethnicity, needs to be explored further.

To attend to the issues reviewed above, there are many ways in which privacy and anonymity can be integrated into the intervention but, at the same time, still provide support to participants, thereby increasing acceptance of and adherence to the interventions, as it normally the case with face-to-face mental health services. One of such ways is adding to the existing Internet-based mental health interventions e-mail and telephone support from therapists, which was shown to lead to favorable mental health outcomes and higher completion rates (e.g., Klein et al., 2009; Mohr, Vella, Hart, Heckman, & Simon, 2008). Additionally, several studies, including Andersson et al. (2005) and Clarke et al. (2005), reported higher intervention adherence and greater reduction of depressive symptoms that resulted from adding automated reminders such as postcards, telephone or email reminders into their Internet-based self-help programs for depression. While the main function of these automated messages is to remind participants to complete exercises or assessments integrated into the e-mental health interventions—that is, taken at their face value, these reminders are not preconceived to be psychological or therapeutic in nature—they still may serve as feedback mechanisms which convey that the participants are actively involved in the program.

Another key factor when developing culturally relevant e-mental health interventions is exploring the learning styles of Asian American women as users of e-mental health tools. Although none of previous empirical studies investigated the relationship between Asian American women’s type(s) of learning styles and their increased use of e-mental health tools, general studies involving both men and women showed that e-health literacy has a strong positive impact on the acceptance of e-mental health interventions (Chang & Chang, 2004; Khazaal et al., 2008). More specific to the type of learning styles, in a study of both male and female users of an online anxiety program, Al-Asadi, Klein, and Meyer (2014) found that those participants who learned best by reading, hearing, and doing were more committed to and motivated by the program than those who learned best by looking and watching.

### Determining Appropriate Program Length

Our second recommendation for the design and development of e-mental health interventions is to consider the length of the intervention study. Given that dropout rates increase with an increase of the length of the program (Christensen, Griffiths, Groves, & Korten, 2006), program length is an important factor to consider. For instance, a study of a 12-week online program for depression (which targeted the general population) showed that time was the greatest barrier to completing the required activities within the program (Crisp & Griffith, 2014). More specifically, Crisp and Griffiths (2014) revealed that even though the participants’ high level of psychological distress were correlated with an increased adherence to a program that would be helpful for them, the participants also reported limited availability in completing tasks within the program due to their busy schedules and perceptions that the program was long. Therefore, in order to increase adherence without compromising the effectiveness of the program, Crisp and Griffiths (2014) recommended shortening the length of the intervention (which was considered to be longer than that of other related online interventions). What the results of this study illustrate considerations of the length of a specific program are particularly relevant when designing e-mental health interventions for Asian American women. Therefore, further research on the ways to reduce length of intervention programs while not reducing their envisaged efficacy is warranted. One approach to address this issue is to ensure that the ease and convenience of use, perceived helpfulness of the intervention, and the likelihood of future use of the intervention are accounted for in determining the length of the program at stake (Crisp & Griffiths, 2014). In this respect, what should be prioritized is not the actual length of the program, but rather the features of the intervention that would increase engagement of the participants.

### Conducting Feasibility Studies

Our third recommendation for the design and development of e-mental health interventions is to conduct feasibility studies that would explore acceptability and adherence of an intervention prior to determining preliminary efficacy of that intervention. Said differently, before integrating efficacy testing in the design and development of e-mental health interventions, it is imperative to determine whether the planned intervention is useful and usable in the first place.

Processes that facilitate testing the acceptance of and adherence to e-mental health intervention include determining which demographic variables would be significant predictors to acceptance and adherence, conducting pre-test and post-tests of variables (e.g., satisfaction, usability), and applying experimental designs to compare variables indicating causative factors to acceptance of and adherence to e-mental health interventions (Christensen et al., 2009; Waller & Gilbody, 2009). However, prior to applying these processes, and considering that many previous studies on acceptance of and adherence to e-health intervention programs have a weak theoretical base, it is important to use a theoretical approach when measuring acceptance, adherence, and dropout. One such theoretical approach to determine acceptance of and adherence to e-mental health interventions recommended by Christensen et al. (2009) is the health belief model (Rosenstock, Strecher, & Becker, 1988). This model is based on two main components that affect an individual’s decision to seek help, namely, (1) the threat of illness; and (2) the expectations of treatment (Rosenstock et al., 1988). Accordingly a person’s desire to prevent illness and his/her belief that a particular health action will prevent or treat an illness are strong predictors of help-seeking behaviors. In a study that applied the health belief model to explore Asian Americans’ help-seeking behaviors toward mental health services, Kim and Zane (2016) found that Asian Americans with increased psychological distress had lower intentions of seeking mental health services than their White American counterparts.

Another relevant theoretical framework to investigate acceptance of and adherence to e-mental health interventions among Asian American women is the theory of planned behavior (Ajzen, 1985). This theory asserts that a “person’s intention to perform (or not to perform) a behavior is the immediate determinant of that action” (Ajzen, 1985, p. 12). Prior e-mental health studies applying the theory of planned behavior demonstrated that previous experience with online mental health services is significantly related to the intentions for using e-mental health tools (e.g., March et al., 2018; Sprenger, Mettler, & Osma, 2017).

In sum, using theoretical frameworks would provide a more coherent approach to exploring acceptance of and adherence to e-mental health intervention programs. As noted by Davis and Addis (1999), using a theoretical framework would enable linking particular characteristics of the studied population and the intervention with the phenomena of acceptance and adherence.

Another important factor to consider vis-à-vis increasing acceptance of and adherence to e-mental health intervention research, specifically in the case of Asian American women who frequently hold stigmatized views of mental illness, is the location of participant recruitment. In this respect, Nicholas et al. (2010)—though this study did not focus specifically on Asian Americans—found that participants recruited from clinical settings were less likely to complete the program than those recruited from non-clinical settings, such as through community mental health organizations and through print media. Trying to interpret this intriguing finding, Nicholas and colleagues (2010) speculated that the participants who were recruited in clinics were recently diagnosed and might have needed more time to come to terms with their diagnosis before taking part in an online treatment program. Therefore, in future research, it would also be beneficial to explore whether the recruitment locations would influence Asian American women’s adherence to e-mental health programs.

## Implications for Research

Based on the results of the extensive literature review undertaken in the present study, and deriving from the recommendations we formulated above, we propose a model that defines a framework of the areas to explore when designing and developing e-mental health interventions and intervention protocols (see Figure 1). We also argue that this model is particularly helpful to nurses, who are primary investigators when it comes to exploring the challenges of and barriers to conducting e-mental health research among Asian American women. Since the proposed model takes into account the specificity of the Asian cultural context, adopting it in practice would help nurse researchers to develop culturally-sensitive recruitment and retention strategies for e-mental health intervention programs.

Moreover, our proposed model of exploring challenges in conducting e-mental health intervention research among Asian American women also implies that nurse researchers should consider the uptake of e-mental health interventions in applied healthcare settings during the program development stage. Accordingly, researchers should begin planning the processes and strategies on applying e-mental health intervention by clinicians and other stakeholders for their clients. This also means that iterative designs of the interventions and further research should involve the community and industry stakeholders who would be potential facilitators of testing e-mental health tools. Upon completion of the research and testing phases, the access to and use of e-mental health tools by Asian American women should be considered. In the long run, if the main purpose of developing and testing e-mental health interventions is to increase access to mental health care, what is crucial in this context is proactive planning for the processes of increasing the use of e-mental health resources.

However, the proposed model (see Figure 1) has several limitations. First, the empirical basis of our model derives from the currently limited literature on Asian American women’s use of e-mental health services. The extensive literature review that formed the conceptual foundation of our model was primarily based on two areas of research: the one focused on Asian Americans’ use of mental health services, on the one hand, and e-mental health studies that were not racially and ethnically specific, on the other hand. Therefore, our proposed model (Figure 1) provides a foundational framework in addressing the challenges with conducting e-mental health intervention research among Asian American women. The second limitation of our model is related to the heterogeneity of the Asian culture. While, in the present discussion paper, we tacitly assumed Asian cultures to share many common characteristics (e.g., the concept of “losing face” or the lack of differentiation between the somatic and the mental in conceptualization of disease), Asian subgroups (i.e., Chinese, Filipino, Japanese, Korean, etc.) may have distinct cultural values and beliefs that, in turn, may differentially influence the recruitment of and adherence to e-mental health intervention research among respective cultural/ethnic groups. Therefore, further research on testing the proposed model in the design and development of e-mental health intervention programs for different groups of Asian American women is necessary.

## Implications for Practice

Nurses play a significant role in encouraging Asian American women to participate in e-mental health intervention research. Along with contributing to exploring the barriers to Asian American women’s participation in e-health mental interventions, their major role lies in supporting facilitating factors that would increase Asian American women’s help-seeking behaviors towards the use of e-mental health services. For example, nurses can encourage their Asian American clients to access online services and information by providing Internet-based discharge instructions. Nurses should also take part in online program development and be key stakeholders in these types of undertakings.

Moreover, nurses, particularly those in primary care settings, should promote high-quality e-mental health resources to their clients. To this end, nurses should familiarize themselves with different credible, relevant, and evidence-based resources. They should also educate themselves about the processes pertaining to referral of clients to e-mental health resources, such as computerized or Internet-based cognitive behavior therapy. They may also need to acquire accurate knowledge about e-mental health resources, get relevant training in using some of these e-mental health tools (particularly those that they would be recommending), and ascertain the effectiveness and safety of those tools. Taken together, these strategies will help normalize and demystify non-traditional mental health services. These processes will also facilitate building a cultural infrastructure that would allow Asian American women to gain more confidence in using e-mental health services, come to view these alternative services as credible and helpful, and, eventually, develop resilience in the midst of their challenges with mental health issues (Reyes & Constantino, 2016).

## Conclusions

The results of the present study on the barriers to conducting e-mental health intervention research among Asian American women highlight the significance of the relevance of such interventions to be tested in the cultural context of Asian American women. More specifically, the strong influence of the collectivistic values shared by many Asian cultures plays a key role in Asian American women’s perceptions of e-mental health services. Our results also highlight several other factors that may affect acceptance of and adherence to e-mental health interventions among Asian American women. As a way to address the afore-mentioned issues, we formulate several recommendations for the design and development of e-health mental interventions targeting Asian American women. Alongside with proposing concrete measures that would help minimize dropouts, this paper emphasizes the importance of considering the processes that can be applied by nurses when the intervention is ready for use, particularly in applied healthcare settings.

## Declaration of Conflicting Interests

The author(s) declared no potential conflicts of interest with respect to the research, authorship, and/or publication of this article.

## Endnotes

1. Due to the scarcity of studies on e-mental health among Asian Americans, we explored current evidence on how the severity of Asian American women’s mental health condition influences these women’s utilization of mental health services. Based on the findings, we speculate on their potential adherence to e-mental health intervention research.

2. This study found that being symptomatic during pre-intervention and the severity of baseline symptoms of depression and anxiety were not significant predictors of adherence. Therefore, these mixed findings with regard to the relationship between the severity of mental health condition and adherence require further investigation.

3. This meta-analysis of previous studies on computerized cognitive behavior therapy has shown no significant relationship between therapist contact time and mental health symptom outcomes.
